# The medical student stressor questionnaire (MSSQ): validation and reliability in Turkish students

**DOI:** 10.1186/s12909-025-07689-4

**Published:** 2025-08-02

**Authors:** Funda Seher Özalp Ateş, Elif Akçay, Derya Gökmen, Süheyla Rahman

**Affiliations:** 1 Department of Biostatistics and Medical Informatics, School of Medicine, Manisa Celal Bayar University, Manisa, Turkey; 2 Department of Child and Adolescent Psychiatry, University of Health Sciences, Ankara Bilkent City Hospital, Ankara, Turkey; 3 Department of Biostatistics, School of Medicine, Ankara University, Ankara, Turkey; 4 Department of Medical Education, School of Medicine, Manisa Celal Bayar University, Manisa, Turkey

**Keywords:** Medical education, Stress, Scale

## Abstract

**Background:**

Medical training is a highly stressful environment for students. In previous studies, many instruments have been used to measure stress levels among medical students. However, these stress measurements are not specific tools for medical settings. This study aims to adapt the Medical Student Stressor Questionnaire (MSSQ) to Turkish, conduct validity and reliability studies, and provide a practical tool to evaluate medical school-related stressors of medical students in Turkey.

**Methods:**

All students studying at the (Manisa Celal Bayar University) School of Medicine were invited to the study. The data was collected between January-March 2024. The research was completed with 412 students. The MSSQ consists of 40 items and 6 subscales. The psychometric properties of the MSSQ were evaluated through tests for validity and reliability. Using RStudio version 1.3.1093 (R Studio, PBC) with the lavaan package, confirmatory factor analysis (CFA) with the “diagonally weighted least squares (DWLS)” estimator was conducted. Internal consistency was tested by Cronbach’s alpha coefficient and test-retest reliability was examined by intraclass correlation coefficient (ICC).

**Results:**

A total of 412 students participated in this study. The median (minimum–maximum) age of the students was found to be 20.00 (18.00–26.00) years. CFA was performed and the appropriate modification indices suggested by the model were added to the model. All 40 items loaded 0.38 or higher. The fit indices calculated as a result of the CFA were determined to be Root Mean Square Error of Approximation (RMSEA) = 0.060 (95% CI: 0.057–0.064)(*p* < 0.001), Comparative Fit Index (CFI) = 0.899 and Tucker-Lewis Index (TLI) = 0.888. The fit indices were at acceptable level. In the analyses conducted for the subscales, the Cronbach Alpha Coefficients were adequate.

**Conclusion:**

In conclusion, this study has provided preliminary evidence for the validity and reliability of a Turkish version of the MSSQ. The six-factor structure was confirmed by CFA. We propose using the Turkish version of MSSQ to evaluate the stress level of medical students. Therefore, further research is needed to investigate the relationship between MSSQ and the other scales which used to measure stress.

**Supplementary Information:**

The online version contains supplementary material available at 10.1186/s12909-025-07689-4.

## Background

Medical training is a highly stressful environment for students [1]. Stress levels are known to be high among medical students during their training and have a negative impact on the well-being of medical students [2, 3]. Previous studies indicate that mental health is particularly impacted, with increased levels of anxiety, depression, and perceived stress among medical students when compared to non-medical student peers [3–5]. Medical students’ prevalence of mental health-related problems, such as stress, anxiety, and depression ranged from 21 to 56% [1, 6, 7]. Moreover, a recent meta-analysis underlined that medical students face several personal, environmental, and academic challenges that may put them at risk for suicidal ideation and suicide attempts [8]. Recent studies continue to confirm these trends across diverse contexts. For instance, Quek et al. found that approximately one in three medical students globally experience anxiety [9]. In Egypt, approximately 93% of medical students experienced moderate to high levels of stress [10]. Although an optimal stress level enhances students’ learning ability, excessive stress can lead to negative consequences, such as concentration problems and low academic performance in medical students [2, 11].

Stress is a subjective experience that results from complex interactions between individuals and their environment [12]. Stress among medical students can arise from various sources, including a demanding academic curriculum, clinical responsibilities, long shifts in emergency care, separation from family, and examination systems [13–17]. Among these, academic stressors are particularly significant contributors to the overall stress experienced by medical students [13, 14]. Additionally, major psychosocial stressors are feelings of loneliness and challenges in relationships with the opposite sex [13, 15]. Furthermore, food quality provided in mess halls or at home is an important environmental factor that significantly predicts stress levels [13, 16]. While stress among medical students is a global concern, country-specific stressors can be shaped by cultural, educational, and systemic factors. In Turkey, the centralized and highly competitive medical education system, combined with a heavy curriculum load and post-graduation uncertainty due to obligatory service and specialty exams (e.g., TUS exams), creates a unique stress profile among Turkish medical students. Research among Turkish medical students suggests that stress and burnout have become increasingly prevalent in the final years of medical education [18–20]. Identifying stressors in medical students is important to reduce increased stress and preventive measures for mental health problems in medical school settings [21].

In previous studies, many instruments have been used to measure stress levels among medical students, such as The Perceived Stress Scale (PSS), Depression, Anxiety, and Stress Scale-21 (DASS-21) [11, 15, 22–24]. However, these stress measurements are not specific tools for medical settings. Additionally, these instruments do not specifically examine the origin of medical students’ stresses. For example, a qualitative study finds that Turkish medical students’ main concerns include the need to develop quickly and adapt to a demanding study environment, having very little time for social life, arts, sports, and entertainment due to their intensive curriculum, as well as aggression and violence directed at health professionals [25]. MSSQ was developed to identify sources of stress in medical students based on the literature, expert opinions, and several stress models [26]. MSSQ identifies various sources of stress in medical students, which are categorized as follows: academic, interpersonal, teaching and learning, social, drive/desire, and group activity. It is a self-reporting and self-scoring tool that requires students to assess the intensity of stress caused by each potential source on a scale from 0 to 4 [21, 26]. It has been validated in many countries, such as Malaysia [27], the Netherlands [28], Romania [29], Nepal [30], India [31], and Sri Lanka [32]. A more recent study from Italy has also confirmed the cross-cultural applicability and strong psychometric properties of the MSSQ [33]. In Turkey, there is no measurement tool to determine the stressors of medical students. There is currently no version of the MSSQ that has been adapted to Turkish. Thus, this study aims to adapt the MSSQ to Turkish, conduct validity and reliability studies, and provide a practical tool to evaluate medical school-related stressors of medical students in Turkey.

## Methods

All students who were studied at the (Manisa Celal Bayar University) School of Medicine were invited to the study. The participants of the study were medical students aged 18 years or older. The voluntary nature of participation was clearly stated in the invitation to the study. At the beginning of the online survey form, students were informed about the aim and scope of the study. They were also informed of their right to decline participation or to withdraw from the study at any time without providing a reason. Participants provided explicit informed consent by selecting the option “I agree to participate in this study” before accessing the questionnaire. No participant was included without completing this step. Data were collected anonymously via Microsoft Forms; no personally identifiable information (e.g., names, student ID numbers, IP addresses) was collected or stored. The data was collected between January-March 2024. The study protocol was approved by the Ethics Committee of the Faculty of Manisa Celal Bayar University (Date/no: May 3, 2023/20.478.486/1816). All procedures performed in this study involving human participants were conducted in accordance with the Helsinki Declaration. For the adaptation of the MSSQ to Turkish, permission was obtained from Dr.Yusoff, who developed the scale, through e-mail.

In the process of adapting the MSSQ into Turkish, the first step involved conducting studies to ensure language validity. Three academicians proficient in both languages translated the original scale from English to Turkish. Following a consensus meeting, these translations were merged into a single version. During this meeting, discrepancies in wording or meaning among the translated versions were discussed, and decisions were made through mutual agreement to ensure semantic and conceptual equivalence. Cultural appropriateness and clarity for the target population were also considered in finalizing the items.

Then, the Turkish form of the scale was back-translated to English by one academician (different from the academicians who had translated it from English to Turkish) who had not seen the English form of the scale previously and known both languages and cultures well. The original and back-translated versions were compared to identify potential inconsistencies, which were resolved in consultation with the research team.

The pilot study of the scale was conducted with 22 students to evaluate the intelligibility of the questionnaire. It was shown that the statements in the questionnaire were easily understandable by students. Although no specific question was included to directly assess item clarity, none of the participants reported any difficulty in interpreting the items, and no feedback indicating a need for revision was received. It is stated in validation studies that the number of participants must be at least 5 and ideally 10 times the number of items on the scale so that factor analysis could be conducted [34, 35]. As the number of items in the MSSQ Scale is 40, we aimed to reach 400 participants.

The research was completed with 412 students. In evaluating the reliability of the scale, the MSSQ was re-applied to 68 of 412 students 4–6 weeks after the scale was administered.

The MSSQ was developed by Yusoff et al. [26]. The MSSQ consists of 40 items and 6 subscales: First subscale is “Academic Related Stressors (ARS)”, second subscale is “Intrapersonal and Interpersonal Related Stressors (IRS)”, third is “Teaching and Learning Related stressors (TLRS)”, fourth is “Social Related Stressors (SRS)”, fifth is “Drive and Desire Related Stressors (DRS)” and sixth subscale is “Group Activities Related Stressors (GARS)”. The items are five point Likert type (0: causing no stress at all, 1: causing mild stress, 2: causing moderate stress, 3: causing high stress and 4: causing severe stress). Each sub-dimension score is obtained by dividing the sum of the items in the relevant sub-dimension by the number of items. Yusoff et al. [26] found that the MSSQ is a valid and reliable instrument that can be used to identify students’ stressors as well as measure the intensity of the stressors. MSSQ has a high internal reliability for total (Cronbach’s α = 0.95) and sub-dimensions (Cronbach’s α is 0.921 for ARS, 0.895 for IRS, 0.858 for TLRS, 0.710 for SRS, 0.646 for DRS, 0.728 for GARS). They conducted their studies with 761 medical students.

There are 13 items in “Academic Related Stressors (ARS)” subdimension. ARS consists of various academic-related stressors, including examination systems, assessment and grading methods, academic schedules, and student activities related to academic events. These stressors may involve receiving poor marks in exams, setting high expectations for academic success, handling large amount of study material, having difficulty to understand content, lack of time to do revision, learning context full of competition, and having difficulty to answer questions given by teachers. A high score in this subdimension reflects that academic-related issues are the main sources of stress.

“Intrapersonal and Interpersonal Related Stressors (IRS)” has 7 items. Interpersonal and intrapersonal related stressors refer to any form of relationships between and within individuals that cause stress.

Intrapersonal stressors are internal and often involve personal struggles, such as low motivation to study or self-conflicts. On the other hand, interpersonal stressors arise from interactions with others and can include verbal, physical, or emotional abuse, as well as conflicts with colleagues, teachers, staff, or other personnel. A high score in this subdimension reflects that personal-related issues are the main sources of stress.

The third subdimension which named “Teaching and Learning Related Stressors (TLRS)” has 7 items. Teaching and learning related stressors refer to any events related to teaching or learning that causes stress. These stressors typically involve the relevance and appropriateness of tasks assigned by teachers, the competency of teachers in supervising and instructing students, the quality of feedback provided, the level of recognition and support offered to students, and the clarity of the learning objectives communicated. A high score in this domain indicates that teaching and learning activities are the main sources of stress, reflecting an environment that may be perceived as unsupportive or unfriendly to students. Addressing this issue requires examining various components of the teaching and learning process to identify the factors causing stress for students.

There are 6 items in “Social Related Stressors (SRS)” subdimension. Social-related stressors are stress-inducing factors arising from community and societal interactions. These typically involve challenges such as balancing leisure time with family and friends, working with the public, maintaining private time for oneself, dealing with interruptions from others, and managing patients’ problems. A high score in this domain indicates that social and community events are the main sources of stress, indirectly indicating that students may struggle to engage effectively in social and community activities.

The fifth subdimension which named “Drive and Desire Related Stressors (DRS)” has 3 items. Drive and desire-related stressors stem from internal or external forces that shape a person’s attitudes, emotions, thoughts, and behaviors, ultimately leading to stress. These stressors often involve a lack of motivation to study medicine, influenced by factors such as pursuing the field against one’s will, making an ill-informed choice about the course, losing motivation after realizing the reality of medicine, studying medicine to fulfill parental expectations, or following friends *to* study medicine. A high score in this subdimension indicates that drive and desire are the main sources of stress.

The last sub-dimension, named “Group Activities Related Stressors (GARS)”, consists of 4 items. Group activity-related stressors arise from events and interactions within group settings that contribute to stress. It generally relates to participation in group discussions, group presentations and others expectations to do well. A high score in this subdimension indicates that group activities and interactions are the main sources of stress.

To assess the sociodemographic characteristics of the students who participated in the study, data were collected on their age, gender, year of study, place of residence, and reasons for choosing the school of medicine.

### Data analyses

The psychometric properties of the MSSQ were evaluated through tests for validity and reliability. Data analyses were carried out using RStudio version 1.3.1093 (R Studio, PBC) [36]. Using the lavaan package, confirmatory factor analysis (CFA) with the “diagonally weighted least squares (DWLS)” estimator was conducted [37]. Items with factor loadings above 0.30 were examined as salient. Model fit was evaluated using the comparative fit index (CFI; >0.90 acceptable, >0.95 excellent), Tucker–Lewis index (TLI; >0.90 acceptable, >0.95 excellent), root mean square error of approximation (RMSEA; < 0.08 good, < 0.05 excellent) [38]. As the chi-square/degrees of freedom (df) ratio is between 2 and 5, it can be interpreted as an indicator of good fit [39]. Internal consistency was tested by Cronbach’s alpha coefficient and test-retest reliability was examined by intraclass correlation coefficient (ICC), based on a two-way mixed effects model with consistency type [40]. The validity and reliability of each subscale was also evaluated using Average Variance Extracted (AVE) and Composite Reliability (CR). To assess discriminant validity, the Heterotrait-Monotrait ratio (HTMT) was calculated for each pair of subscales. For each student, sum scores of subdimensions were created where the factor loading of each item is multiplied to the scaled score for each item before summing. The relationships between demographic variables and the subdimension sum scores of the MSSQ were analysed via SPSS 11.5 (SPSS, Chicago, IL, USA). The differences between demographic variables in MSSQ sub-dimension scores were examined using the Mann-Whitney U Test/Kruskal-Wallis Test. Associations between age and MSSQ subdimensions scores were determined by Spearman correlation analysis. A value of *p* < 0.05 was considered statistically significant.

## Results

This research was conducted with students at Manisa Celal Bayar University School of Medicine. Students were included only if they agreed to participate in the study. A total of 412 students (150 Grade I, 104 Grade II, 33 Grade III, 44 Grade IV, 33 Grade V and 48 Grade VI) participated in this study. The median (minimum–maximum) age of the students was found to be 20.00 (18.00–26.00) years. One hundred and seventy five (42.5%) of the students stay in the student dormitory, 146 (35.4%) of them stay in the student house, and 91 (22.1%) of them stay in the family house. The majority of students (*n* = 177, 43.0%) stated that they chose medical school because of their dream of becoming a doctor. Demographic information for total sample (*n* = 412) and test–retest sample (*n* = 68) were given in Table 1.

**Table 1:** Demographic information for total sample and Test-Retest sample

	**All Sample**	Test-Retest Sample
	(n = 412)	(n = 68)
Age		
Median (Min-Max)	20.00 (18.00–26.00)	20.00 (18.00–25.00)
Mean ±SD	20.52 ± 2.01	20.07 ± 1.96
Gender		
Male	170 (41.3%)	24 (35.3%)
Female	242 (58.7%)	44 (64.7%)
The place to stay		
Student dormitory	175 (42.5%)	37 (54.4%)
Student house	146 (35.4%)	17 (25.0%)
Family house	91 (22.1%)	14 (20.6%)
Reason for choosing medical school		
Dream of becoming a doctor	177 (43.0%)	36 (52.9%)
My family wants me to study medicine	36 (8.7%)	4 (5.9%)
My exam score is high	52 (12.6%)	9 (13.2%)
Being a doctor is a prestigious profession	82 (19.9%)	8 (11.8%)
Having good financial income	31 (7.5%)	5 (7.4%)
All of the above	2 (0.5%)	-
Other reason	32 (7.8%)	6 (8.8%)

Min: Minimum, Max: Maximum, SD: Standard Deviation

### Validity

CFA was performed and the appropriate modification indices suggested by the model were added to the model. Correlations were allowed between the errors of the items within the same sub-dimension (item1 with item4 and item25; item2 with item24; item3 with item5, item26 and item31; item4 with item25; item5 with item26 and item39; item9 with item26 and item39; item10 with item33; item11 with item13; item12 with item25; item14 with item20; item25 with item27; item 26 with item28, item31, item39; item28 with item39). The items and factor loadings were given in Supplementary material 1, Table 2. All 40 items loaded 0.38 or higher. The factor loading values at the subscales level ranged between 0.53 and 0.72 for the ARS subscale, between 0.62 and 0.88 for the IRS, between 0.61 and 0.70 for the TLRS, between 0.38 and 0.64 for the SRS, between 0.38 and 0.66 for the DRS and between 0.40 and 0.76 for the GARS subscales. Also, the total contribution of six factors to the variance was 39.59%. The fit indices calculated as a result of the CFA were determined to be RMSEA = 0.060 (95% CI: 0.057–0.064)(*p* < 0.001), CFI = 0.899 and TLI = 0.888. The chi-square/df ratio was 2.51. The fit indices were at acceptable level. Cultural differences in interpreting items could affect the model fit, particularly in cross-cultural studies where certain items might be understood differently across groups. The interpretation of psychological constructs can vary across cultures, which may influence participants’ responses.

The AVE values for each subscale were as follows: ARS (0.39), IRS (0.55), TLRS (0.42), SRS (0.25), DRS (0.29), and GARS (0.39), respectively. Although some AVE values were below the recommended threshold (0.50), all original items were retained to preserve content validity. The HTMT values ranged from 0.629 to 0.844 across the six subscales. Specifically, HTMT values were between 0.776 and 0.844 for ARS, 0.629–0.776 for IRS, 0.656–0.844 for TLRS, 0.747–0.843 for SRS, 0.629–0.831 for DRS, and 0.656–0.815 for GARS. All HTMT values remained below the conservative threshold of 0.85, indicating adequate discriminant validity between the subscales.

The differences between the groups for sub-dimensions were given in Supplementary material 2, Table 3.

There was significant difference between class according to the ARS, TLRS, SRS and GARS score (*p* < 0.001, *p* = 0.015, *p* = 0.001 and *p* < 0.001, respectively). The ARS scores of the students who studied in the third grade were higher than those who studied in the first grade. TLRS scores of the students who studied in the third grade were higher than those who studied in the first grade. The SRS scores of the students who studied in the third grade were higher than those who studied in the sixth grade. The GARS score of the students who studied in the third grade were higher than those who studied in the first, second or sixth grade. The ARS, IRS, TLRS, SRS and GARS scores were significantly higher in females than that in males (*p* < 0.001, *p* < 0.001, *p* = 0.027, *p* < 0.001 and *p* < 0.001, respectively). There was no significant difference between male and female according to the DRS score (*p* = 0.567). The students who stay at a student dormitory had higher SRS score than those who stayed at student or family house (*p* = 0.032). According to the reasons for choosing a medical school, there was a difference only in DRS scores (*p* = 0.027). The DRS scores of students whose parents wanted them to study medicine were higher than those of students who had dream of becoming a doctor or whose exam score was high or who thought that being a doctor was prestigious.

There were no statistically significant correlation between age and ARS, IRS scores (for ARS: *r* = 0.091, *p* = 0.066; for IRS: *r* = 0.066, *p* = 0.179). There were a weak positive correlation between age and TLRS, DRS, GARS scores (respectively, *r* = 0.119, *p* = 0.016; *r* = 0.111, *p* = 0.025; *r* = 0.118, *p* = 0.016). There was a weak negative correlation between age and SRS scores (*r*=-0.105, *p* = 0.033).

### Reliability

Internal consistency (Cronbach alpha coefficient) analysis was used to determine the reliability of the MSSQ-Turkish version. In the analyses conducted for the subscales, the Cronbach Alpha Coefficient was found to be 0.872 for the ARS subscale, 0.823 for the IRS subscale, 0.811 for the TLRS subscale, 0.639 for the SRS subscale, 0.490 for the DRS subscale and 0.697 for the GARS subscale. For the test-retest reliabilities, the ICC (95% Confidence Interval) values were 0.740 (0.610–0.831) for ARS, 0.791 (0.682–0.866) for IRS, 0.618 (0.447–0.746) for TLRS, 0.567 (0.381–0.709) for SRS, 0.515 (0.317–0.670) for DRS and 0.714 (0.574–0.813) for GARS. The CR values for each subscale were as follows: ARS (0.89), IRS (0.90), TLRS (0.83), SRS (0.66), DRS (0.54), and GARS (0.71), respectively.

## Discussion

This study aimed to adapt the Turkish version of the MSSQ and evaluate its validity and reliability, an internationally recognized scale for assessing stress level for a representative sample of Turkish medical school students. This study provides initial evidence supporting the validity and reliability of the Turkish version of the MSSQ. The Sri Lankan and Italian version of the MSSQ had five factors [32, 33]. The Italian version had 39 items. The Romanian version had six factors with 20 items [29]. The goodness-of-fit statistics for the six-factor structure in this study were found to be adequate. All 40 items loaded 0.38 or higher. The subscales and their items were similar to those in the other versions of MSSQ. The differences in factor structures could be a result of cultural differences. Different societies and cultures may not provide the same structure. In this study, the six-factor structure with 40 items was suitable and aligned with the original version of scale. In our study, Cronbach’s alpha coefficient was 0.94 for the MSSQ, 0.87 for ARS, 0.82 for IRS, 0.81 for TLRS, 0.64 for SRS, 0.49 for DRS and 0.70 for GARS. The DRS and SRS subscales showed relatively low internal consistency. Similar findings have been reported in other MSSQ adaptation studies, suggesting that certain items may be interpreted differently depending on cultural or institutional context [31, 32]. While the subscales were retained to preserve the original factor structure and allow cross-study comparisons, further research is needed to assess the cultural relevance of individual items and explore potential refinements for improved reliability.

These findings show that the Turkish version of the MSSQ has adequate internal consistency. Moreover, the results of our study confirmed that the scale has a adequate test-retest reliability.

In the validity and reliability study conducted with Italian students, Cronbach’s alpha value of the scale was found to be 0.94 (vary between 0.67 and 0.89 for subscales) [33]. In a Sri Lankan study, Cronbach’s alpha of the scale was found to be 0.95 and Cronbach’s alpha of the subscales ranged from 0.54 to 0.90 [32]. Recently, for Romanian students, Cronbach’s alpha has been found to vary between 0.70 and 0.90 for subscales and 0.88 for the total scale [29]. In Indian study, the Cronbach’s alpha coefficient was found to be above 0.8 for the total scale and ARS subscale. For IRS, TLRS and GARS, the Cronbach’s alpha coefficient ranged from 0.5 to 0.8, but for SRS and DRS were less than 0.5 [31]. These findings show that the DRS subscale has demonstrated weak reliability not only in the Turkish sample but also in other cultural contexts, particularly in the Indian and Sri Lankan versions. This suggests that the psychometric weakness of the DRS subscale may reflect broader cross-cultural differences rather than a country-specific issue.

The variation in the internal consistency of the DRS subscale across different cultural adaptations of the MSSQ may be explained by several context-specific factors. Cultural norms influence how medical students perceive and express ambition, expectations, and future concerns—key elements measured by the DRS. For instance, in collectivist cultures, career-related stress may be closely linked to family or societal obligations, while in individualistic societies, personal achievement may be emphasized more directly [41, 42]. Additionally, differences in the structure and competitiveness of medical education systems can shape the relevance of such stressors [4]. Linguistic nuances in the translation of related terms of “drive/desire,” despite careful back-translation, may lead to variation in how these constructs are understood [43]. Furthermore, cultural response styles, such as socially desirable responses or central tendency bias, can impact how consistently students respond to DRS items, thereby affecting reliability scores [44]. The lower reliability of the DRS subscale in Turkey may be related to the cultural tendency to express personal ambition and future expectations less openly. Additionally, the motivation to pursue medical education is often shaped more by societal or familial expectations than by individual desire, which may lead to inconsistent responses to DRS items [42]. When considered together, these factors provide a plausible explanation for the recurrently low internal consistency coefficients reported for the DRS subscale in different cultural settings.

In this study, while Composite Reliability (CR) values for all subscales ranged from 0.54 to 0.90, indicating acceptable internal consistency, Average Variance Extracted (AVE) values varied between 0.25 and 0.55. Although some AVE values were below the recommended threshold of 0.50, this is not uncommon in early-stage scale adaptation studies. All original items were retained to preserve the theoretical integrity and content validity of the scale. Furthermore, acceptable CR and ICC values support the internal consistency and temporal stability of the Turkish version of the instrument.

The all HTMT values remained below the commonly accepted threshold of 0.85; however, the value for the SRS subscale (0.843) was very close to the cutoff.This suggests potential conceptual overlap between some subscales, which may slightly limit the discriminant validity of the instrument. Further item-level analyses may help clarify these relationships in future studies.

Although the fit indices obtained from the CFA fall within the acceptable range, the CFI and TLI values are close to the lower bound of recommended thresholds. This may reflect cultural and educational differences that influence how certain stressor items are perceived by Turkish medical students. For example, differences in clinical exposure, curriculum structure, or academic expectations could lead to varying interpretations of item content. While the original factor structure was retained to maintain comparability with the original MSSQ, future research may consider refining or rephrasing specific items to enhance model fit.

As most participants were early-year students, some stressors commonly experienced during clinical years— such as patient-related stress, time pressure during rotations, and emotional fatigue —may be underrepresented. This limits the generalizability of the results to more advanced student groups. Future studies including a more balanced distribution across academic years are needed to further validate the tool’s applicability to clinical-year students.

The MSSQ may enhance awareness of factors (gender, class, the place to stay, reason for choosing medical school) which can influence level of stress in medical school students. Consistent with the study done by Ghosal and Behera [45], girls had higher stress level compared with boys. In this study, the ARS, IRS, TLRS, SRS and GARS scores were significantly higher in girls than in boys. The students who stayed “student dormitory” had higher SRS score than stayed in “family” or “student” house. The DRS scores of students whose families wanted them to study medicine were found to be higher than those of students who chose medical school because “they dreamed of becoming a doctor” or “they had high exam scores” or “they thought medicine was a prestigious profession”. Examining at the sub-dimensions, in general, the scores of 3rd grade students are higher than other students. In Malaysian study, the grade was only significant factor of stress level [27]. In the study on prevalence of stress in medical students, academic stress was found to be significantly more in medical students of 2nd year. This difference found here may be due to differences in course curricula between countries [45].

The Turkish adaptation of the MSSQ provides valuable insights into the stressors experienced by medical students in Turkey. This tool can be effectively used in educational and clinical settings to identify and address the specific stress factors affecting students’ well-being. For instance, faculty members and student support services can utilize the MSSQ to assess stress levels in students and tailor interventions accordingly, such as stress management workshops, counseling services, or curricular adjustments to alleviate identified stressors.

Furthermore, the MSSQ can be a useful tool in longitudinal studies, tracking changes in stress levels throughout medical education and helping to identify critical periods where students may be at higher risk of burnout or mental health challenges. By incorporating the MSSQ into regular student assessments, universities and medical institutions can better support students in managing academic stress, leading to improved academic performance, well-being, and overall mental health.

In addition, MSSQ results can be used to inform institutional decision-making processes such as modifying course loads in particularly high-stress semesters, optimizing exam schedules, or introducing targeted mentoring for vulnerable groups. Data gathered from MSSQ assessments may also serve as evidence to guide the development of institutional mental health strategies and national medical education policies aimed at fostering a more supportive learning environment.

### Limitations and future research

This study had some limitations. The relationship between MSSQ and other scales that used for measuring stress levels could not be examined. This restricts the evaluation of the tool’s convergent and discriminant validity. Without such comparisons, it is difficult to determine whether the scale uniquely measures the intended construct and how it relates to theoretically similar or distinct concepts. Future research should aim to include validation with comparable tools to strengthen the evidence for construct validity.

Although the six-factor model accounted for 39.59% of the total variance, this may be attributed to the broad scope of the MSSQ and the multidimensional nature of stress among medical students. Cultural and contextual differences in item interpretation may also have contributed to the relatively lower explained variance.

A further limitation is the relatively low internal consistency of the DRS and SRS subscales, likely due to their limited item numbers. Future research may consider item refinement or alternative measures to enhance reliability.

The majority of the students participating in this study were Grade-1 and Grade-2. This limit the generalizability of the findings to clinical-year students. An increase in stress levels could be observed in medical students, especially after they start working in the clinic, that is, after Grade-4. Clinical students are likely to encounter different stressors, including direct patient care, time management challenges in clinical rotations, and greater emotional burden, which might affect the stressor profile captured by the MSSQ. The distribution of students to grades is unbalanced in our data set. This uneven distribution may introduce bias, as stress levels can differ substantially between pre-clinical and clinical phases. As a result, the generalizability of our findings to all medical students should be interpreted with caution. Future studies should aim to ensure more balanced representation across academic years. Since there is a need for studies on examining stress level in Turkish medical students, this scale in studies will significantly contribute to the literature.

## Conclusion

In conclusion, this study provides preliminary evidence supporting the validity and reliability of the Turkish version of the MSSQ. The six-factor structure of the scale was confirmed through confirmatory factor analysis, and the instrument appears suitable for assessing stress levels among Turkish medical students. Notably, gender was found to have a significant effect on MSSQ scores. This may be partially explained by culturally embedded gender roles and expectations within Turkish society, where female students may face additional stressors, such as balancing academic demands with social or familial responsibilities. These dynamics could contribute to elevated stress levels in specific subgroups. Therefore, future research should explore the underlying causes of gender-related differences in stress responses and examine the relationship between MSSQ scores and other validated stress-related instruments.

## Electronic supplementary material

Below is the link to the electronic supplementary material.

**Supplementary Material 1** (The Items and Factor Loadings (Standard Error) (for Turkish version)).

**Supplementary Material 2** (Comparisons of the Groups for Sub-dimensions).

## Data Availability

The dataset collected and analysed during the current study is available from the corresponding author upon reasonable request.

## Abbreviations

MSSQ: Medical Student Stressor Questionnaire
CFA: Confirmatory Factor Analysis
DWLS: Diagonally Weighted Least Squares
ICC: Intraclass Correlation Coefficient
RMSEA: Root Mean Square Error of Approximation
CFI: Comparative Fit Index
TLI: Tucker Lewis Index
ARS: Academic Related Stressors
IRS: Intrapersonal and Interpersonal Related Stressors
TLRS: Teaching and Learning Related Stressors
SRS: Social Related Stressors
DRS: Drive and Desire Related Stressors
GARS: Group Activities Related Stressors
df: Degrees of Freedom
AVE: Average Variance Extracted
CR: Composite Reliability
HTMT: Heterotrait-Monotrait Ratio
Min: Minimum
Max: Maximum
SD: Standard Deviation
CI: Confidence Interval
